# The Evolution of Gene Expression QTL in *Saccharomyces cerevisiae*

**DOI:** 10.1371/journal.pone.0000678

**Published:** 2007-08-01

**Authors:** James Ronald, Joshua M. Akey

**Affiliations:** Department of Genome Sciences, University of Washington, Seattle, Washington, United States of America; Indiana University, United States of America

## Abstract

Understanding the evolutionary forces that influence patterns of gene expression variation will provide insights into the mechanisms of evolutionary change and the molecular basis of phenotypic diversity. To date, studies of gene expression evolution have primarily been made by analyzing how gene expression levels vary within and between species. However, the fundamental unit of heritable variation in transcript abundance is the underlying regulatory allele, and as a result it is necessary to understand gene expression evolution at the level of DNA sequence variation. Here we describe the evolutionary forces shaping patterns of genetic variation for 1206 *cis*-regulatory QTL identified in a cross between two divergent strains of *Saccharomyces cerevisiae*. We demonstrate that purifying selection against mildly deleterious alleles is the dominant force governing *cis*-regulatory evolution in *S. cerevisiae* and estimate the strength of selection. We also find that essential genes and genes with larger codon bias are subject to slightly stronger *cis*-regulatory constraint and that positive selection has played a role in the evolution of major *trans*-acting QTL.

## Introduction

Gene expression is the primary intermediate between information encoded by the genome and higher order phenotypes, and as a result expression variation is thought to be an important source of phenotypic diversity. Considerable effort has been devoted to characterizing patterns of natural variation in and the evolutionary trajectories of gene expression levels both within and between species [1]–[9]. A reoccurring observation in these studies is that transcript abundance varies considerably, with a significant amount of this variation attributable to heritable genetic changes that affect gene expression levels in a quantitative manner [10]. A powerful paradigm to emerge in studies of gene expression variation has been the combination of microarray technology and genetic mapping, allowing many gene expression QTL to be identified [11]–[19]. Because these QTL point to regulatory polymorphisms that are the underlying units of heritable variation in transcript abundance, understanding the forces governing their evolution can provide detailed insights into gene expression diversity within populations and divergence between species.

To study the evolutionary forces acting on regulatory polymorphisms, we took advantage of a large, well-studied data set of gene expression QTL discovered between the *S. cerevisiae* laboratory strain BY4716 (BY, isogenic to S288C) and the wild vineyard strain RM11-1a (RM) [11], [20]–[24]. We leveraged the available whole genome sequences of BY, RM, the clinical isolate YJM789 (YJM) [25], and the outgroup *Saccharomyces paradoxus* [26] to make inferences about the evolutionary forces acting on DNA sequence variation underlying regulatory QTL. Furthermore, we made use of the known ancestral history of S288C to identify a key recent departure from mutation-purifying selection-drift equilibrium in the regulatory program of laboratory yeast.

Our analyses represent a first step toward applying population genetics models to a large set of QTL underlying variation in functional genomics phenotypes. As it becomes feasible to collect these data in large and cosmopolitan samples within a species, population genetics approaches and evolutionary modeling will become increasingly important and informative. Finally, although we focus on gene expression levels, our approaches provide the conceptual foundation for understanding the evolutionary forces shaping extant patterns of variation in other genetically tractable functional genomics phenotypes.

## Results

### Genomic distribution of regulatory QTL

**Figure 1:**
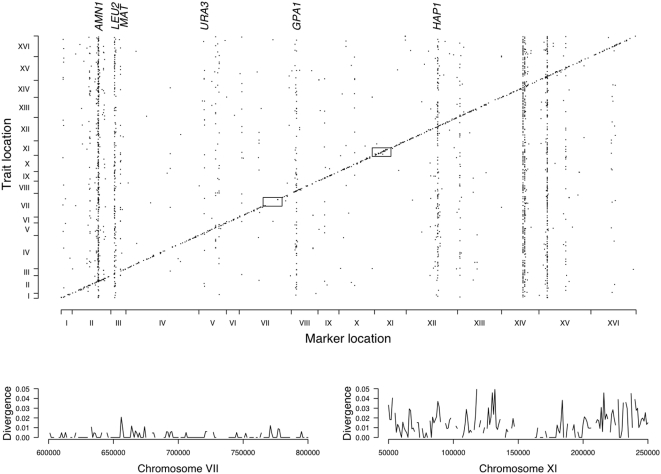
Genomic distribution of regulatory QTL between BY and RM. Location of the gene whose expression level is under inspection (vertical axis) versus marker location (horizontal axis) for 2368 trait marker pairs (points) with significant linkage at an experiment-wide permutation based FDR≤0.05. Identities of known major *trans*-acting QTL are listed above. Lower panels show the synonymous site substitution rate for the two chromosomal regions indicated by boxes. Breaks in the curves are due to the absence of synonymous sites in intergenic regions. As previously described, regions with low neutral substitution rates contain fewer *cis*-acting QTL [23].

In segregants derived from a cross between the BY and RM strains, a large number of gene expression levels show significant linkage to markers throughout the genome [11], [20], [23]. Figure 1 shows the location of 2368 genes (out of 5067 total) that demonstrate linkage at a false discovery rate (FDR) ≤0.05 (see Text S1). Vertical bands indicate single major *trans*-acting QTL that influence large numbers of gene expression levels throughout the genome. The diagonal band represents QTL that are located coincident with the gene under inspection. We previously used allele-specific expression measurements and comparative sequence analysis to show that the majority of these QTL are due to *cis*-acting polymorphisms in the promoter and 3′ UTR of the corresponding gene [23]. In this paper, we refer to genes whose expression levels show linkage coincident with their own location as *cis*-acting QTL and, although there appears to be a minor role for local *trans*-acting polymorphisms at these loci, we refer to the causative polymorphisms at these loci as *cis*-acting regulatory polymorphisms. In addition, our models are constructed to account for the contribution of nearby *trans*-acting QTL that occur on the same chromosome as the expression trait of interest, thus producing linkage that mimics *cis*-acting QTL. For convenience we refer to genes whose expression levels fail to show significant linkage to their own loci as genes without *cis*-regulatory variation, although in reality a sizable fraction of these genes are expected to harbor undetected *cis*-regulatory polymorphisms due to the incomplete power of the data set (see below). In the analyses described below we focus primarily on *cis*-acting QTL because each represents an independent evolutionary event, they are more abundant than *trans*-acting QTL (1206 *cis*-acting QTL, see Text S1, versus approximately 100–200 *trans*-acting QTL [20]), are detected more reliably [19], and in contrast to *trans*-acting QTL their locations are known with more precision. Finally, it is important to emphasize that in contrast to an observed DNA polymorphism, a QTL is an estimated quantity, defined by statistically significant linkage between a trait and a particular genomic region. As a result, our models are constructed to account for the uncertainty inherent in the QTL detection process. Furthermore, we show that our estimates of key parameters are robust to various QTL detection thresholds.

### Rate of accumulation of *cis*-acting QTL

A commonly used strategy to detect deviations from neutrality is to compare the rate of accumulation of putatively functional changes (e.g. non-synonymous substitutions) to putatively neutral changes (e.g. synonymous substitutions). Using this approach, we compared the observed rate of accumulation of *cis*-acting QTL to what is expected under neutrality. Loci throughout the genome show different levels of neutral substitution between BY and RM due to ancestral recombination (see Text S1), so if *cis*-acting QTL are selectively neutral, we expect them to accumulate at a clock-like rate based on their locus-specific coalescence time. Assuming that there are (on average) *n* regulatory sites per gene and that the coalescence time at locus *i* is *t_i_* (measured in units of *N_e_* generations), then the probability that a gene shows *cis*-acting expression variation is the probability that any regulatory site undergoes mutation
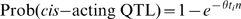
(1)where θ = 2μ*N_e_*. If some fraction, δ, of genes do not tolerate or have lost regulatory polymorphisms due to purifying selection, then the probability that a randomly chosen gene shows *cis*-acting expression variation is
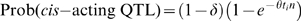
(2)For each locus the divergence time can be estimated via the synonymous substitution rate (see Methods and Text S1), but the precise value of the locus-specific divergence time is unknown. Therefore, we compute the joint probability of observing *m_i_* substitutions at *M_i_* neutral sites and a *cis*-acting expression change at locus *i* by integrating over the full range of coalescence times, weighted by their probability density function *e^−t^*

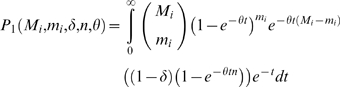
(3)Similarly, the joint probability of observing *m_i_* substitutions at *M_i_* neutral sites and no *cis*-acting expression change at locus *i* is given by
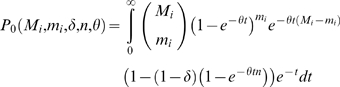
(4)Combining Equations 3 and 4 and the power, Prob(linkage|*cis*-acting QTL) (estimated to be 0.504, see Methods), and false positive rate, Prob(linkage|no *cis*-acting QTL) (estimated to be 0.039, see Methods), gives the likelihood of the pattern of genes showing significant linkage to *cis*-acting QTL
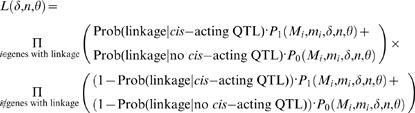
(5)The neutral model (δ = 0) is nested in the parameter space at the boundary (0≤δ≤1). Therefore, twice the difference between the log*_e_* likelihoods of the purifying selection model and the neutral model is distributed as a 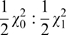 distribution.

**Figure 2:**
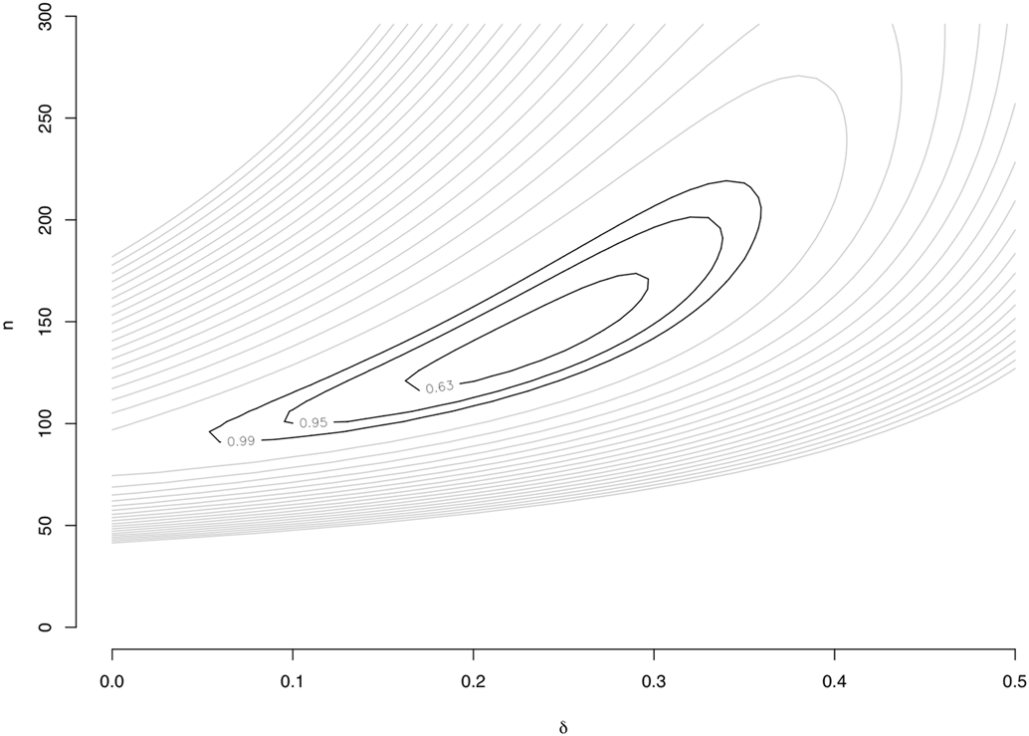
Likelihood surface for δ and *n*. Black contours show the 63%, 95%, and 99% confidence intervals for the joint value of δ and *n* (based on the χ^2^_2_ distribution with Δ log*_e_* likelihood = 1, 3, and 5, respectively). Gray contours correspond to Δ log*_e_* likelihood = 10, 15, 20, …, 100.

**Figure 3:**
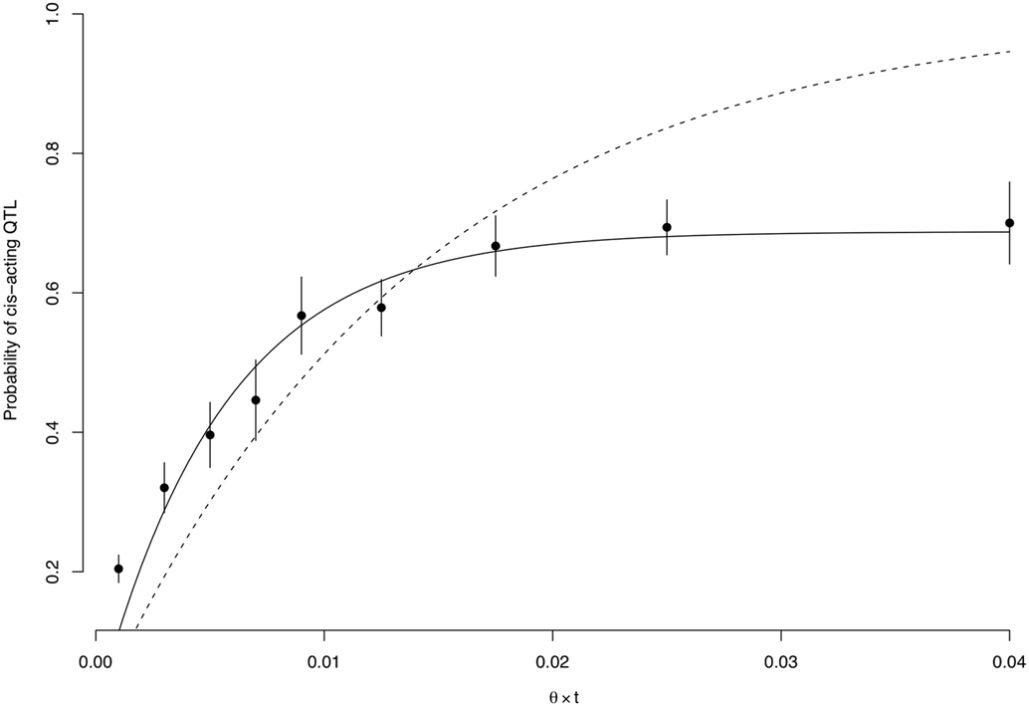
Rate of accumulation of *cis*-acting QTL. Genes were divided into bins based on their locus-specific maximum likelihood estimate of θ×*t_i_*. The rate of *cis*-acting QTL in each bin (points, 95% CIs shown in vertical lines) was estimated based on the observed number of genes with linkage and the estimated power and false positive rate of linkage analysis. The least squares fit of the purifying selection model to the points (solid line) results in estimates of δ and *n* of 0.31 and 181 which are somewhat larger but not significantly different from the estimates obtained under the likelihood based approach. The dashed line shows the least squares fit of the neutral model, yielding *n* = 71.

Using this approach, the maximum likelihood estimates of δ, *n*, and θ were 0.24 (95% CI 0.13–0.32, Figure 2), 144, and 0.009, with the purifying selection model representing a significant improvement over the fully neutral model (*p* = 1.2×10^−4^) where δ was constrained to zero and the estimates of *n* and θ were 85 and 0.009. Other QTL detection thresholds yielded quantitatively similar estimates (see Text S1). Note that an estimate of 144 regulatory sites per gene under the purifying selection model, although perhaps large, is in reasonable agreement with the observations that polymorphisms in both the promoter and 3′ UTR contribute to regulatory variation [23], that approximately 40% of intergenic sites in *S. cerevisiae* are subject to purifying selection [27], and that numerous sequence features appear to contribute to message stability [28]–[30]. To provide a more intuitive and graphical representation of the models, we fit Equations 1 and 2 to the data by a simple regression based alternative to the likelihood approach as shown in Figure 3 (see Methods).

We considered other possible explanations for the poor fit of the neutral model relative to the purifying selection model, including inability to detect linkages for some genes due to low expression, variation in the number of regulatory sites per gene, and microarray hybridization artifact, but these alternative models appear to be less plausible than the purifying selection model (see Text S1). It is important to note that the estimate of δ was sensitive to the estimated power to detect *cis*-acting QTL by linkage analysis and to a lesser extent to the estimated false-positive rate. This sensitivity is to be expected, since the power and false-positive rate determine how well the observed pattern of significant linkages captures the true underlying pattern of *cis*-acting regulatory variation. However, as described in Text S1, further analyses suggest that our estimate of the power is conservative making the estimate of δ an underestimate.

### Allele frequency distribution of *cis*-regulatory polymorphisms

The estimated value of δ does not imply that 24% of genes are without *cis*-acting regulatory variation. Instead, this estimate reflects that regulatory evolution is proceeding slower than the neutral prediction and that at any given level of divergence, approximately 24% fewer genes show *cis*-acting expression variation than would be expected if these expression changes were selectively neutral. This deficiency could be due to strong purifying selection against expression changes in a subset of genes, persistent weak purifying selection against expression changes in most genes, or a combination of these possibilities.

**Figure 4:**
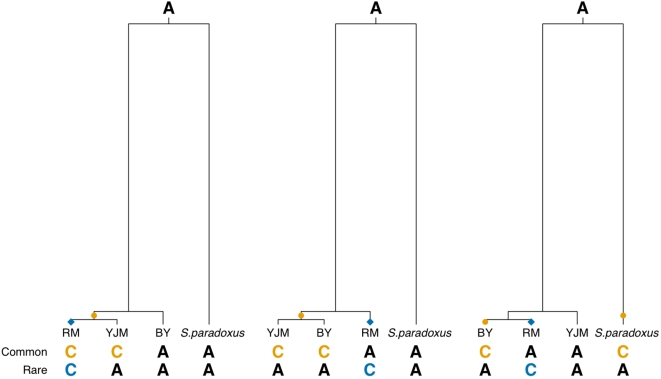
Illustration of common and rare derived alleles between BY and RM. The three possible rooted tree topologies for the *S. cerevisiae* strains are shown with branch lengths approximately to scale. Hypothetical genotypes for a polymorphism between BY and RM are given below. Orange and blue points represent mutations between BY and RM that result in common and rare derived alleles, respectively. Note that repeat mutation leads to apparently common derived alleles as illustrated for the right most topology.

In order to understand the relative contributions of these processes, we evaluated the allele frequency distribution of existing regulatory polymorphisms segregating in 932 out of the 1206 genes with *cis*-acting regulatory variation for which we could identify orthologs in BY, RM, and YJM (which can be regarded as a randomly mating, recombining population [31], [32]; see also Figure S1 and Text S1) and the outgroup *S. paradoxus*. We determined whether the frequency distribution of *cis*-acting regulatory polymorphisms was skewed toward rare derived alleles, which tend to be recent and occur in sites otherwise conserved in both *Saccharomyces* lineages. Such an approach has previously been used as an indicator of weak purifying selection [33]-[35]. We classified derived alleles between BY and RM as rare if either the BY or RM allele was observed in both YJM and *S. paradoxus* or as common if both the BY and RM alleles were observed in YJM and *S. paradoxus* (Figure 4).

We found that polymorphisms in the key *cis*-acting regulatory regions of these genes (the promoter region from 101 to 200 bases upstream of translation start [23], [36], [37] and the 3′ UTR from 1 to 100 bases downstream of translation stop [23]) were skewed toward rare derived alleles relative to synonymous site polymorphisms in the same 932 genes, consistent with the action of purifying selection (Table 1). Approximately 68% of promoter and 3′ UTR derived alleles between BY and RM in genes with *cis*-acting regulatory variation are classified as rare compared to 61% of synonymous derived alleles (Fisher's exact test, *p* = 9.0×10^−5^). This skew drops off in intergenic regions around the consensus yeast promoter and is absent in more distal downstream intergenic regions (Table 1). In addition, we found that for promoter polymorphisms the skew toward derived alleles was larger in genes with *cis*-regulatory changes, suggesting that purifying selection acts more strongly to restrict large perturbations in gene expression (Figure S2).

**Table 1:** 

	932 genes with *cis*-regulatory variation		932 nearby genes without *cis*-regulatory variation		Remaining 2352 genes without *cis*-regulatory variation	
Region	Percent rare (number)	Fisher's exact *p*-value (versus)	Percent rare (number)	Fisher's exact *p*-value	Percent rare (number)	Fisher's exact *p*-value
Promoter	69% (371)	5.8×10^−4^ (synonymous)	63% (256)	0.058	70% (430)	3.5×10^−9^
3′ UTR	67% (361)	0.044 (synonymous)	65% (249)	0.0074	68% (399)	3.4×10^−6^
Upstream intergenic	65% (617)	0.24 (promoter and 3′ UTR)	62% (530)	0.47	67% (857)	0.21
Downstream intergenic	62% (425)	0.018 (promoter and 3′ UTR)	62% (414)	0.58	65% (615)	0.051
Non-synonymous	70% (836)	0.24 (promoter and 3′ UTR)	71% (787)	0.0023	76% (1464)	1.1×10^−4^
Synonymous	61% (1756)	NA	58% (1491)	NA	58% (2314)	NA

Skew in the frequency distribution of derived *cis*-acting regulatory alleles. The percentage and number of all derived alleles that are rare are shown for each region for 932 genes with *cis*-regulatory variation, 932 nearby genes without *cis*-regulatory variation, and the remaining 2352 genes without *cis*-regulatory variation. Promoter refers to 101–200 bases upstream of translation start. 3′ UTR refers to 1–100 bases downstream of translation stop. Upstream intergenic is 1–300 bases upstream of translation start excluding the promoter region. Downstream intergenic is 101–300 bases downstream of translation stop. Within each set of genes, statistical tests compare the proportion of rare alleles between the indicated region versus the region listed in parentheses.

For comparison, we performed the same analysis on 932 genes without linkage to *cis*-acting QTL located within 5 kb of each of the genes with *cis*-regulatory variation to approximate the same distribution of tree topologies and branch lengths in the two sets of genes (Table 1). We also performed the analysis for 2352 genes without *cis*-regulatory variation located elsewhere in the genome. Although there is a significant excess of rare derived alleles in the promoter and 3′ UTR relative to synonymous sites in these genes, some skew is to be expected given our power to detect *cis*-regulatory effects is incomplete. In addition, it is likely that some of these polymorphisms lead to *cis*-acting expression variation between BY and RM under other growth and environmental conditions, and a skew toward rare derived alleles is consistent with the action of weak purifying selection on such environmentally dependent regulatory sites. Interestingly, the skew towards rare alleles is more extreme for both intergenic and non-synonymous changes in the set of 2352 genes located distant from detected *cis*-acting QTL. The explanation for this effect is presumably due to the shallower genealogies across these 2352 loci, in which the mean number of changes per synonymous site was 0.0067 as compared to 0.012 in the 932 genes with *cis*-regulatory variation and the 0.011 in the 932 adjacent genes without *cis*-regulatory variation. These shallower genealogies imply approximately half the level neutral variation, and hence a higher ratio of mildly deleterious to neutral changes at these loci as evidenced by the significantly elevated rare derived allele skew in non-synonymous changes.

In spite of the skew toward rare derived alleles in putative regulatory regions of genes without statistically significant *cis*-regulatory variation, it is notable that the effect is significantly less extreme than for non-synonymous changes in these genes. In genes with detectable *cis*-regulatory variation, derived alleles show a skew similar to non-synonymous changes, suggesting that the regulatory mutations we detect may be associated with fitness costs commensurate with non-synonymous mutations.

### Modeling the allele frequency skew under the ancestral selection graph

**Figure 5:**
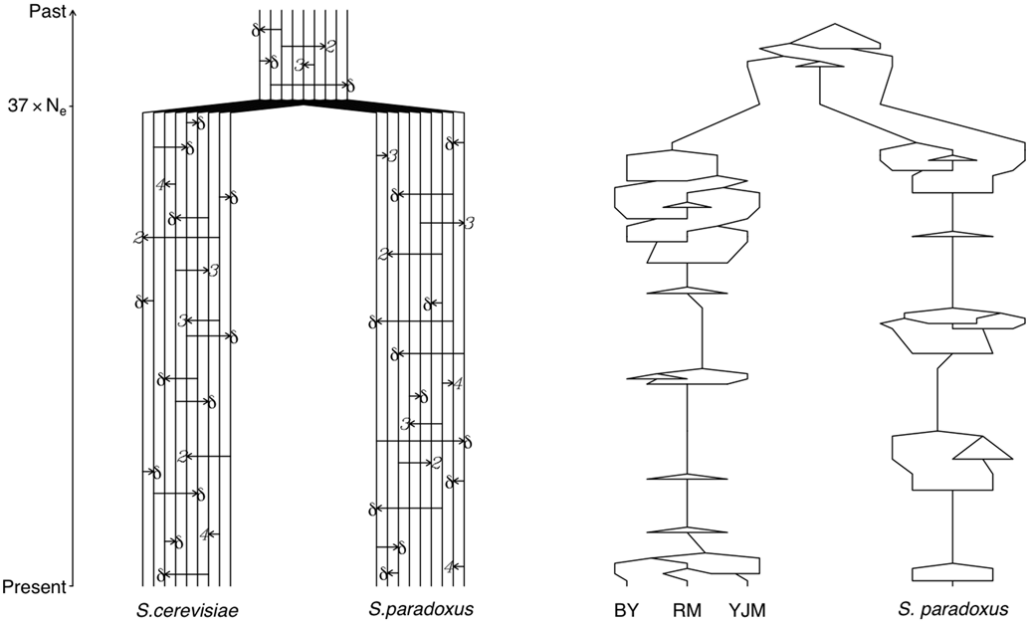
Ancestral selection graph simulation scheme. The left panel shows a percolation diagram illustrating the underlying Moran model with neutral births realized by all individuals (δ arrows) and extra births realized only by fitter individuals (*2*, *3*, and *4* arrows) (see [38], [39] for detailed discussion). The right panel shows a realization of the reverse time simulation process for four sampled individuals representing the three *S. cerevisiae* strains and *S. paradoxus*. After mutations have been placed on the graph, branching events are resolved depending on the fitness of the two potential ancestors. Resolution of branching events produces a typical coalescent tree but introduces a bias towards advantageous alleles.

**Figure 6:**
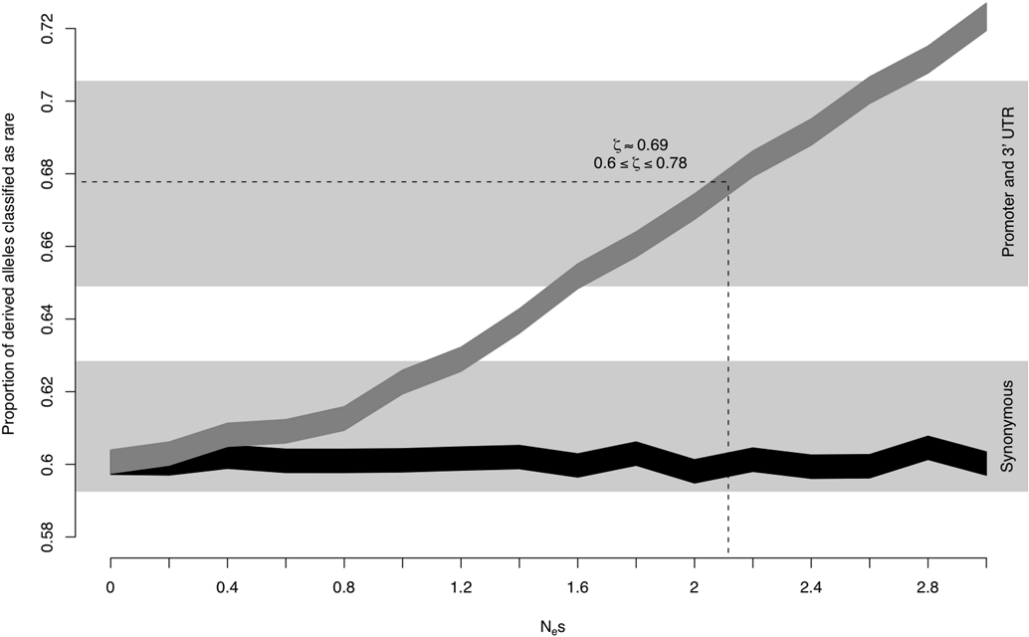
Strength of purifying selection against *cis*-acting regulatory changes. Light gray shaded areas indicate that 95% CIs for the proportion of rare derived alleles (vertical axis) in synonymous sites and in the promoter and 3′ UTR. Ninety-five percent CIs for the expected proportion of rare derived alleles at selected sites (dark gray shading) and linked neutral sites (black shading) are shown as a function of the scaled fitness difference between selective classes (horizontal axis). The dashed line indicates the scaled purifying selection coefficient (2.1) that is most likely to have produced the observed allele frequency skew based on linear interpolation between 2*N_e_s* = 2.0 and 2.2. The rate of substitution at the selected site relative to the linked neutral site, denoted by ζ, is indicated along with the 95% CI.

To estimate the strength of selection giving rise to the observed rare derived allele skew, we performed simulations under the ancestral selection graph, an extension of the coalescent that incorporates natural selection [38], [39] (see Methods and Figure 5). Each simulation included a selected site (with fitness values in terms of the selection coefficient 2*N_e_s*) representing a *cis*-regulatory site and a linked neutral site representing a synonymous site. For each realized genealogy in the simulations, if the two sampled individuals representing BY and RM were polymorphic with respect to each other at the selected (or neutral) site, we determined whether the derived allele was common or rare as defined above. Figure 6 shows the average proportion of rare derived alleles at the selected and neutral sites as a function of the scaled fitness difference between selective classes. The value of the scaled fitness difference between selective classes which best fits the observed skew for regulatory polymorphisms is 2.1 (95% CI based on the binomial distribution approximately 1.6–2.7). Note that this is likely an underestimate because in the observed data some synonymous sites may not be selectively neutral and because the magnitude of the observed allele frequency skew may be diminished by neutral polymorphisms in the promoter region and 3′ UTR that do not affect regulatory sites.

These simulations also allowed us to ask at what rate mildly deleterious alleles are lost between BY and RM. Comparing the number of times the selected site was polymorphic between BY and RM to the number of times the linked neutral site was polymorphic, we estimate that with a scaled fitness difference between selective classes of 2.1, the substitution rate at selected sites relative to neutral sites (analogous to *d_I_*/*d_S_* , the intergenic regulatory substitution rate to synonymous substitution rate and denoted by ζ [40]) was 69% (95% CI 60%–78% based on the 95% CI for 2*N_e_s*; see Methods). We considered additional selection models (see Text S1) and found that under these other models estimates of ζ were at most 0.73. If the substitution rate at regulatory sites is approximately 0.7 times the neutral substitution rate, then the probability that a gene shows *cis*-acting regulatory variation would be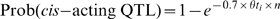 Note that this differs from Equation 2 because in analyzing the rate of accumulation of *cis*-acting QTL it is not possible to estimate both the number of regulatory sites and a mutation rate multiplier at these sites simultaneously. Both models are similar, however, in that they describe a slower rate of accumulation of *cis*-acting QTL relative to neutrality. Using the estimated values of *n* and θ obtained above, the expected deficiency in the rate of *cis*-acting QTL across all loci would be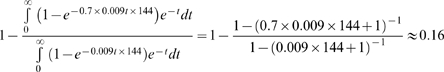 Therefore, based on the mutation-weak purifying selection-drift equilibrium predicted by the patterns of genetic variation apparent at existing *cis*-acting QTL, we predict there should be 16% fewer such QTL than under neutrality. Above, we estimated a 24% deficiency in the number of *cis*-acting QTL based on their rate of accumulation across all genes. Thus, the strength of purifying selective acting on extant *cis*-acting polymorphisms would be expected to produce nearly the same deficiency in the number of *cis*-acting QTL observed across all genes.

### Features associated with slower *cis*-regulatory evolution

As described above, weak purifying selection appears to be sufficient to explain much of the deficiency in *cis*-acting QTL. Nonetheless, in order to determine whether certain classes of genes were subject to stronger selective pressure against expression changes than others, we determined whether any particular features were associated with *cis*-acting expression changes. There was a slight but significant deficiency in the number of essential genes among genes with *cis*-regulatory variation (16% versus 20% in genes lacking *cis*-regulatory variation, Fisher's exact test, *p* = 0.0034). There was also significantly less codon bias among genes with *cis*-regulatory variation compared to genes without (mean codon bias 0.095 versus 0.125, Wilcoxon rank test, *p* = 3.4×10^−4^). We did not find a significant difference in tolerance to amino acid change (comparing *d_N_*/*d_S_* among *Saccharomyces sensu stricto* species [26] in genes with versus without *cis*-regulatory variation, data not shown) suggesting that structural constraint does not necessarily predict gene expression constraint.

### Evolution of major *trans*-acting regulatory QTL

In contrast to our estimates of a 16%–24% deficiency in the number of *cis*-acting QTL, approximately 94% of genes show heritable expression variation between BY and RM (see Text S1), with many linking to major *trans*-acting QTL as shown in Figure 1. If most individual expression changes (as mediated by *cis*-acting polymorphisms) are mildly deleterious, then we expect that the cumulative selective effects against major *trans*-acting QTL would be so strong that they would be rapidly eliminated from the population. The existence of such major *trans*-acting regulatory QTL therefore suggests that alleles at these loci may confer some selective benefit to mitigate the deleterious effects on gene expression. There are two examples of such QTL that support this hypothesis. The first is the trivial case of the *leu2*Δ*0* allele (Figure 1), which experienced selection in the laboratory as an auxotrophic marker. The second is *AMN1* in which the D368V loss of function mutation in BY is responsible for widespread gene expression changes [20], [23]. Loss of function of *AMN1* also causes cellular dispersal in BY, rather than the clumpy growth observed in RM [20]. BY is derived from the strain S288C, and as described by Robert Mortimer, “Conditions established for this strain [S288C] were that it be nonclumpy (nonflocculent) - i.e., dispersed as single cells in liquid culture…” [41]. We sequenced *AMN1* in the available S288C natural isolate progenitor strains EM93, EM126, NRLL YB-210, and “Yeast Foam” [41] (which account for approximately 95% of the S288C ancestry) and found that they possess the aspartate allele at residue 368 rather than valine, suggesting that this novel major *trans*-acting regulatory allele is unlikely to be found in the wild but instead was fixed during Mortimer's selection for cellular dispersal. Thus, the phenotypic benefits of leucine requirement and cellular dispersal in the laboratory apparently facilitated the emergence of widespread gene expression changes, which might otherwise be intolerable to the cell in the wild.

## Discussion

By analyzing a large set of *cis*-acting QTL discovered between divergent yeast strains, we have provided an initial description of the evolutionary forces acting on gene expression QTL. We have shown that *cis*-acting QTL accumulate more slowly than expected under neutrality and that the underlying regulatory polymorphisms are skewed toward rare derived alleles. Thus, weak purifying selection against expression polymorphisms appears to be a pervasive force acting on gene expression levels for yeast in log phase growth in rich media. We estimated a scaled selection coefficient of ≈2 for typical *cis*-regulatory changes, indicating that selection, though detectable, is rather weak with stochastic forces playing a significant role in shaping patterns of within population *cis*-regulatory diversity. Under such a nearly neutral regime, unpreferred *cis*-regulatory alleles are expected to be present at appreciable frequencies, and an interplay of forces, including changes in effective population size, linkage disequilibrium among selected alleles, and epistatic selection [42], is expected to figure prominently in *cis*-regulatory evolution over longer time periods.

Given the widespread weak purifying selection on gene expression, the existence of major *trans*-acting regulatory alleles that affect hundreds of genes throughout the genome is surprising. We proposed that deviations from the normal mutation-purifying selection-drift regime may allow such alleles to persist in the population. At these loci, we hypothesized that the deleterious transcriptional effects of novel major *trans*-acting regulatory alleles tend to be balanced by beneficial phenotypic effects of these same alleles.

There are several important caveats to our analyses. First, as described above and in Text S1 our analyses of the rate of accumulation of *cis*-acting QTL were sensitive to the estimated power of the linkage analyses and to a lesser extent to the estimated false-positive rate. As larger expression QTL data sets are collected, better estimates of these quantities can be obtained, allowing for more precise estimation of the extent to which purifying selection affects gene expression QTL. Second, although we interpreted the observed excess of rare derived *cis*-regulatory alleles as further evidence of weak purifying selection, some fraction of these rare alleles may be due to fixation of beneficial regulatory changes within divergent *S. cerevisiae* strains due to positive selection. As described below, we expect that weak selection to maintain gene expression stability may result in positive selection on certain compensatory regulatory changes in spite of the fact that most novel alleles are likely to be deleterious.

Although our analyses were based on a small number of yeast strains, they make several predictions about the pattern of expression QTL that might be observed among strains in the yeast population. First, based on our conclusion that most *cis*-acting QTL are mildly deleterious, we expect that among any pairwise comparison of strains, fewer *cis*-acting QTL would be present than predicted under neutrality. Second, selection against extant *cis*-regulatory alleles is rather weak, we would not expect to observe a set of genes that have invariant *cis*-regulation across numerous yeast strains. Instead, we predict that most or all genes are likely to show *cis*-regulatory polymorphism in the global population of yeast strains. Finally, we provided anecdotal evidence that positive selection may allow major *trans*-acting regulatory QTL to emerge and persist in the population. In addition to positive selection, demographic perturbations such as bottlenecks or population structure may lead to the emergence of novel major regulatory alleles. However, in the absence of such forces, we would expect few major *trans*-acting regulatory QTL. Indeed, in outbred populations such as humans, the existence of major *trans*-acting QTL is controversial (contrast [13] with [14]).

In the broader context of gene expression evolution, recent studies in a variety of species have found widespread signatures of purifying selection in patterns of gene expression variation [1], [5], [7], [43]. Our results provide the first comprehensive evaluation of the evolutionary forces acting upon regulatory QTL and suggest that the evolutionarily stable expression patterns observed at the level of overall transcript abundance are due to persistent weak purifying selection acting against novel regulatory alleles in most genes. Studying gene expression evolution in terms of the underlying regulatory QTL is an important first step towards a more detailed and quantitative understanding of the forces governing regulatory evolution and allows new hypotheses to be explored. For example, if selection acts to constrain gene expression levels to an optimal level but novel regulatory alleles persist with long sojourn times due to weak purifying selection, then compensatory regulatory evolution [44] may be common. Compensatory fixation of additive regulatory alleles with opposing effects would be expected to result high levels of transgressive segregation, consistent with previous observations [45]. In addition to coevolution among regulatory alleles, it has also been suggested that *cis*-regulatory alleles may coevolve with alleles of the associated protein as a mechanism for titrating gene activity [46]. More generally, our results confirm theoretical predictions that mildly deleterious regulatory QTL segregate in natural populations [42] and raise the possibility that these polymorphisms contribute to phenotypic diversity.

## Materials and Methods

### Strains, expression data, and sequence analysis

Strains BY4716, RM11-1a, and YJM789 and *S. paradoxus* have been described elsewhere [11], [20], [25], [26]. Whole genome sequences for BY (isogenic to S288C), RM and *S. paradoxus*, and YJM were obtained from the Saccharomyces Genome Database (http://www.yeastgenome.org/), the Broad Institute (http://www.broad.mit.edu/annotation/fungi/fgi/), and the Stanford Genome Technology Center (http://www-sequence.stanford.edu/ yjm789 public/), respectively. Gene expression measurements and genotypes in 112 segregants in the cross between BY and RM are from Brem and Kruglyak [45]. Whole genome linkage analyses and tests for *cis*-acting regulatory variation are described in Text S1 and in Ronald et al. (2005) [23]. To estimate the locus-specific coalescence time for each gene we created whole chromosome alignments for BY and RM using LAGAN [47] as described in Text S1. We identified orthologous genes by reciprocal best match using CROSSMATCH (http://bozeman.mbt.washington. edu/phrap.docs/phrap.html) and performed alignments of BY, RM, YJM, and *S. paradoxus* genes and intergenic regions using CLUSTALW [48]. We purchased strains EM93, EM126, NRLL YB-210, and “Yeast Foam” from America Type Culture Collection (catalog #204501), Herman J Phaff Culture Collection (#40-126), and Centraalbureau voor Schimmelcultures (#6333 and #1428), respectively. Primer sequences for *AMN1* were 5′-CCAAAGGAAAGACCATGCTT-3′ and 5′-CTAGCGCGACCAGTGAGAC-3′.

### Power and false-positive rate to detect *cis*-acting QTL by linkage analysis

Using linkage analysis to detect *cis*-acting QTL involves a false-positive and false-negative rate. In order to estimate these quantities, we must compare the number of statistically significant linkage tests with the estimated number of truly null and truly alternative linkage tests. We must also account for apparent linkages to *cis*-acting polymorphisms which occur instead because of linked *trans*-acting regulatory genes that are on the same chromosome but distinct from the locus in question. Such QTL are problematic for the model because their locations, and hence their locus-specific coalescence times, are unknown. To estimate these quantities, we used the method of Storey and Tibshirani to analyze the complete distribution of *p*-values to estimate the overall proportion of truly alternative tests [49].

First, we estimated the proportion of truly null tests (denoted π_0_) and truly alternative tests (1−π_0_) across all 5067 single marker linkage tests performed at the marker closest to the locus of the gene in question. For this set of tests, π_0_≈0.514 suggesting that approximately 2464/5067 of tests are truly alternative in the sense that a regulatory QTL affecting the expression level of the gene in question is linked to the marker locus. These regulatory QTL include both the *cis*-acting QTL that we are interested in as well as linked *trans*-acting QTL on the same chromosome. Assuming that *trans*-acting regulatory QTL are distributed randomly with respect to their target genes, we estimated the rate at which these single marker linkage tests detect linked *trans*-acting regulatory QTL by testing each gene expression trait for linkage to randomly chosen markers in the genome [23]. The rate at which regulatory QTL are detected among these tests provides an estimate of the genome-wide prevalence of polymorphic *trans*-acting regulatory loci between BY and RM. The estimate of π_0_ for this set of tests is approximately 0.898, indicating that about 518/5067 of these single marker linkage tests at random loci detect true *trans*-acting QTL. Thus,


	Prob(*cis*-acting QTL ∪ linked *trans*-acting QTL) = 
       	Prob(*cis*-acting QTL) + Prob(linked *trans*-acting QTL)
       	- Prob(*cis*-acting QTL) · Prob(linked *trans*-acting QTL) ≈ 2464/5067

	Prob(linked *trans*-acting QTL) ≈ 518/5067
	Prob(*cis*-acting QTL) ≈ 0.428


In order to estimate the rate of true *cis*-acting QTL among the set of genes with statistically significant linkage, we must subtract out two types of false signals: linkages to nearby *trans*-acting QTL as described above and statistical false-positives in which neither a *cis*-acting nor a linked *trans*-acting QTL exists, but instead the gene expression level shows a spurious correlation with the marker. The rate at which statistical false-positives occur can be estimated by the FDR. For the 1206 linkage tests significant at LOD≥1.37, the FDR estimated by Storey's and Tibshirani's method was 0.026. Thus, approximately 1175 out of these 1206 significant tests are expected to be truly alternative in the sense that a *cis*-acting or *trans*-acting QTL is linked to the marker in question. There were 157 linkages called significant at LOD≥1.37 among the 5067 random marker linkage tests, and the associated FDR was ≈0.342. Thus, approximately 103 true-positive *trans*-acting QTL are detected in the 5067 random single marker linkage tests. Using these estimates the rate at which true *cis*-acting QTL are called significant can be calculated as follows


	Prob(significant linkage∩(*cis*-acting QTL ∪ linked *trans*-acting QTL)) = 
       Prob(linkage∩*cis*-acting QTL)+Prob(linkage∩linked*trans*-acting QTL)
       – Prob(linkage∩*cis*-acting QTL) ·Prob(linkage∩linked *trans*-acting QTL)≈1175/5067
	Prob(significant linkage∩linked *trans*-acting QTL)≈103/5067
	Prob(significant linkage∩*cis*-acting QTL)≈0.216


The estimated power to detect *cis*-acting QTL is then Prob(significant linkage∩*cis*-acting QTL)÷Prob(*cis*-acting QTL)≈0.504 and the estimated false-positive rate is (Prob(significant linkage)-Prob(significant linkage∩*cis*-acting QTL))÷(1-Prob(*cis*-acting QTL))≈0.039. As described in Text S1, we expect that these estimates are conservative.

### Regression based approach for the rate of accumulation of *cis*-acting QTL

As described above, the probability that a gene shows *cis*-acting regulatory variation depends on the coalescence time *t_i_* between the two copies of the gene. For each locus, the value of *t_i_* is unknown, but it is straightforward to obtain a point estimate of *t_i_* based on the observed locus-specific synonymous site substitution rate. If at locus *i* there are *m_i_* synonymous substitutions observed between BY and RM among a total of *M_i_* synonymous sites, then
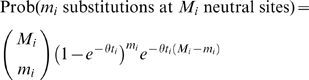
(6)and the maximum likelihood estimate of θ*t_i_* is 
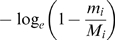
. These locus-specific estimates could then be plugged into Equation 2, yielding
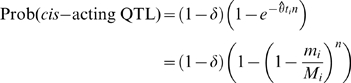
(7)Grouping genes based on the observed 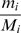 allows for a simple regression of Prob(*cis*-acting QTL) on the estimated θ*t_i_* as a function of the parameters of interest δ and *n*, as given by Equation 7 and as shown in Figure 3.

### Ancestral selection graph simulations

We used the ancestral selection graph to estimate the scaled selection coefficient that would give rise to the observed skew towards rare derived alleles at regulatory polymorphisms. We modeled BY, RM, and YJM as sampled individuals from a common *S. cerevisiae* population with *S. paradoxus* as the outgroup (diverged 37*N_e_* generations ago). This scheme is shown in Figure 5. Each simulation included a neutral site and a linked selected site at which the scaled selection coefficients for the four nucleotides were σ*_T_* = 0, σ*_C_* = 2*N_e_s*, σ*_G_* = 2×2*N_e_s*, and σ*_A_* = 3×2*N_e_s*. Our implementation of the ancestral selection graph applies to evolution in haploid populations or diploid populations in which selection acts additively. Mutation occurred at both loci at a rate of θ/2 along each branch with θ = 2μ*N_e_* = 0.01 (based on the observed synonymous site substitution rate) according to the Kimura two parameter model with a transition to transversion ratio of 4.4 (estimated from the observed data). Note that by allowing mutation and selection to occur continuously in both *Saccharomyces* lineages, we correctly account for the effect of repeat mutations on our parsimony based counts of rare derived alleles under both the null hypothesis (neutral evolution) and the alternative hypothesis (purifying selection). We performed 1×10^7^ simulations for 2*N_e_s* = 0.0, 0.2, 0.4, …, 3.0. We also considered other selection models in which transition mutations lead to a larger decrease in the fitness (see Figure S3 and Figure S4 and Text S1). All other aspects of the ancestral selection graph simulation were performed according to the algorithms outlined in Neuhauser and Krone [38].

At a scaled fitness difference between selective classes of 2*N_e_s* = 2.0 (2.2), there were 69379 (66022) simulations out of 1×10^7^ for which the two sampled individuals representing BY and RM were polymorphic with respect to each other at the selected site. Of these polymorphisms 45427 (44130) were classified as having rare derived alleles whereas 22277 (20507) were classified as common. As in the observed data, unclassified polymorphisms occur because more than two alleles are observed among the four *Saccharomyces* sequences at a single site. The scaled selection coefficient that most closely reproduced the observed skew was estimated to be 2.1 by linear interpolation between 2*N_e_s* equal to 2.0 and 2.2. For 2*N_e_s* = 2.0 (2.2), the linked neutral site was polymorphic 98247 (97017) times (slightly less than 1×10^7^×θ due to repeat mutation), and 52844 (52508) of these polymorphisms were categorized as having rare derived alleles whereas 35513 (34819) were classified as common. This proportion of rare derived alleles is not significantly different from that observed at synonymous sites, supporting the simple demographic model employed in these simulations. The decreased rate of polymorphism between BY and RM at the selected site relative to the neutral site (ζ = 69379/98247≈71% at 2*N_e_s* = 2.0; ζ = 66022/97017≈68% at 2*N_e_s* = 2.2) reflects the elimination of mildly deleterious alleles by purifying selection. Linear interpolation between these values provides an estimate of the deficiency in regulatory polymorphism due to weak purifying selection.

## Supporting Information

Text S1 Supplementary materials describing in detail the methods and results for supporting analyses.
(0.11 MB PDF)

Figure S1 Observed synonymous site substitution rate autocorrelation function (blue points) and 10 realizations of the autocorrelation function from simulated yeast genomes (black lines). We imposed the same pattern of missing data on the simulated data as was present in the observed data (due to gaps, to low quality regions of the alignments, and to the absence of synonymous sites in intergenic regions and because of overlapping ORFs). In both the simulated and observed data the autocorrelation function was calculated for each chromosome and then averaged across the 16 chromosomes.
(0.17 MB TIF)

Figure S2 Magnitude of the skew toward derived alleles as a function of *cis*-regulatory effect size. The proportion of derived alleles classified as rare are shown for synonymous polymorphisms (black), promoter polymorphisms (blue), and 3' UTR polymorphisms (orange) in genes with *cis*-regulatory variation.
(0.11 MB TIF)

Figure S3 Strength of purifying selection against *cis*-acting regulatory changes with Σ_T_ = 0, Σ_G_ = 2N_e_s, Σ_C_ = 2×2N_e_s, and Σ_A_ = 3×2N_e_s. The relative rate of substitution at the selected site relative to the linked neutral site, denoted by ζ, is indicated along with the 95% CI.
(0.85 MB TIF)

Figure S4 Strength of purifying selection against *cis*-acting regulatory changes with Σ_G_ = 0, Σ_T_ = 2N_e_s, Σ_C_ = 2×2N_e_s, and Σ_A_ = 3×2N_e_s. The relative rate of substitution at the selected site relative to the linked neutral site, denoted by ζ, is indicated along with the 95% CI.
(0.84 MB TIF)
