# Supplementary material for: The Evolution of Gene Expression QTL in Saccharomyces cerevisiae
Source: PLoS One. 2007 Aug 1;2(8):e678. doi: 10.1371/journal.pone.0000678 (PMC1925141; doi:10.1371/journal.pone.0000678)
Supplement: Text S1 — Supplementary materials describing in detail the methods and results for supporting analyses. (0.11 MB PDF) [file pone.0000678.s001.pdf]

Supplement for:

The evolution of gene expression QTL in *Saccharomyces cerevisiae*

James Ronald and Joshua M. Akey

Department of Genome Sciences

University of Washington

Seattle, WA 98195

Identification of regulatory QTL

In order to identify regulatory QTL, we performed linkage analysis treating gene expression levels as quantitative traits in a previously described data set consisting of 112 haploid segregants genotyped at 2954 SNP markers [1, 2, 3]. We analyzed a total of 5067 genes, removing 115 genes from the 5182 described in Ronald *et al.* (2005). These 115 genes were excluded because they had fewer than 100 synonymous sites from which we could estimate the locus-specific coalescence times (see below) due to overlap with other annotated ORFs or frameshift mutations in RM.

To identify regulatory QTL shown in Figure 1 we performed non-parametric linkage analysis using `R/qtl` [4, 5]. To determine the critical LOD score associated with a false discovery rate (FDR) ≤ 0.05, we formed a single permutation of the rows of the 112×5067 phenotype matrix relative to the rows of the 112×2954 genotype matrix. This procedure preserves the correlation among gene expression levels and genotypes for the 112 haploid segregants, but breaks the correlation between gene expression levels and genotypes. We then obtained the genome-wide maximum LOD score for each of the 5067 gene expression traits in the permuted data. The minimum LOD score at which the number of genome-wide maximum LOD scores in the permuted

data was less than 5% of the number of genome-wide maximum LOD scores in the observed data gives the critical LOD score (LOD $\geq$ 3.58) associated with FDR $\leq$ 0.05. Note that this procedure provides a conservative estimate of the FDR because it assumes π_0, the proportion of truly null tests, is equal to 1 [6].

To identifiy *cis*-acting QTL, we performed a hypothesis driven non-parametric linkage analysis where we tested each gene expression trait for linkage at the single marker closest to the location of the gene as described in Ronald *et al.* (2005). This approach reduces the multiple testing associated with whole genome linkage analysis and therefore improves power to detect regulatory QTL. The QTL detected by this procedure map to the same locations when multiple markers are used in a multipoint approach; we also found that the majority of such QTL are due to *cis*-acting polymorphisms [3]. Out of the 5067 gene expression traits analyzed, 1206 showed significant linkage at a permutation based FDR $\leq$ 0.05 (LOD $\geq$ 1.37), determined by permuting the rows of the 112 $\times$ 1 phenotype matrix relative to the rows of the 112 $\times$ 1 genotype matrix, for the phenotype and genotype matrices corresponding to each of the 5067 traits. These 1206 significant linkages are a subset of the 1233 described in Ronald *et al.* (2005) (due to the removal of 115 genes as

described above) and a superset of the 549 genes shown in Figure 1 for which the *cis*-acting variant was the strongest regulatory QTL for the trait across the entire genome and was significant at LOD ≥ 3.58.

Locus-specific coalescence times

In order to model the rate of accumulation of *cis*-acting QTL under neutral models and under models with selection, we estimated the locus-specific coalescence times based on the synonymous substitution rate between BY and RM for each gene.

We created whole chromosome alignments for BY and RM using `LAGAN` [7], manually inspected the alignments, and corrected two major inversions, one on chromosome III from 134990 to 143476 and the other on chromosome XIV from 575382 to 599468. We then estimated the locus-specific coalescence time for each gene by counting the number of synonymous substitutions per site within 2 kb surrounding the promoter of each gene, excluding any gene having fewer than 100 synonymous sites in this 2 kb window, $\geq 5\%$ gap characters, or $\leq 95\%$ total sequence identity. These criteria led to the exclusion of the 115 genes described above. Note that 2 kb corresponds

to the typical recombination block size reported previously [8]. This block length is also supported by the observation that the synonymous substitution autocorrelation function (calculated by counting the number of synonymous substitutions per site in 1 kb intervals across the chromosomes) can be closely approximated by simulated data under a recombining coalescent framework (using `ms` [9] and `Seq-Gen` [10]) with block lengths of approximately 1.5 kb to 3.5 kb (Figure S1). Thus, the observed variation in substitution rate is consistent with the pattern produced by ancestral recombination.

Alternative models for the rate of accumulation of *cis*-acting QTL

We considered a number of other alternative models besides purifying selection which might confound our analysis of the rate of accumulation of *cis*-acting QTL.

First, we noted that the estimated fraction of genes subject to purifying selection δ was sensitive to the estimated power to detect *cis*-acting QTL, with lower estimates of the power leading to lower estimated values of δ. For example, if the estimated power was 0.4 rather than 0.504, then the

estimated value of δ was 0.01 ($p = 0.40$). If the estimated power was 0.6 the value of δ was 0.37 ($p = 1.1 \times 10^{-8}$). This sensitivity is to be expected because if the power to detect *cis*-acting QTL is low, the observed pattern of linkages captures little information about the underlying pattern of *cis*-acting regulatory variation. In contrast, if the power is high, then most of the true underlying pattern is captured by linkage analysis. Similarly, if the estimated value of the false-positive rate was much higher, for example 0.1, the value of δ was 0.12 ($p = 0.17$), whereas if the estimated false-positive rate was much lower, for example 0.001, then the estimated value of δ was 0.26 ($p = 1.8 \times 10^{-8}$). Again this sensitivity is to be expected because if the false-positive rate is high, then much of the true underlying pattern of *cis*-acting QTL is obscured by the large amount of noise in the linkage analysis results.

In order to further explore the sensitivity of our analyses to the estimated power to detect *cis*-acting QTL and the false-positive rate, we repeated our analyses under different linkage analysis significance thresholds. At a p-value cutoff of 0.001 (corresponding to an FDR of 0.003), 801 genes showed significant linkage to markers nearest to their own loci. The estimated power and false-positive rate were 0.355 and 0.011, respectively, consistent with the

expectation that these quantities must both be smaller at a more stringent threshold for QTL detection. Using these data, we found that the maximum likelihood estimates of δ was 0.24 with the purifying selection model showing significant improvement over the neutral model ($p = 1.1 \times 10^{-3}$). At a significance threshold of 0.05 (FDR = 0.076) 1700 genes showed significant linkage to their own loci, and the estimated values for the power, false-positive rate, and δ were 0.649, 0.101, and 0.31, respectively, with the purifying selection model showing significant improvement over the neutral model ($p = 3.0 \times 10^{-10}$). Thus, provided that the estimated power and false-positive rate correspond to the significance threshold used for detection of *cis*-acting QTL, our estimate of δ is reasonably robust to variation in these quantities. Conversely, these analyses suggest that our estimate of δ is relatively insensitive to the significance threshold used to detect *cis*-acting QTL, provided that the rates of false-negatives and false-positives are taken into account.

Next we considered the effect of hybridization artifact. It has previously been shown that inter-species (or inter-strain) comparative gene expression studies which utilize an array platform designed with respect to one individual can detect hybridization differences due to polymorphisms in other

individuals relative to the reference, rather than to expression differences in other individuals relative to the reference [11]. These apparent expression differences would map to the locus of the gene in question mimicking *cis*-acting QTL. The probes on the expression arrays (designed with respect to the S288C genome, to which BY is isogenic) are approximately 1 kb in length and the average substitution rate in the coding sequence between BY and RM is approximately 0.005, so we expect hybridization artifact to be less extreme than the effect detected by Gilad *et al.* (2005). Moreover, we suspect that such linkages to such artifactual *cis*-acting QTL would tend to bias our analyses toward the neutral model (as was suggested by Gilad *et al.* in the context of contrasting their support for a purifying selection model of primate gene expression evolution with previous studies supporting a predominantly neutral model [12]) for several reasons. First Gilad *et al.* noted that genes with differential expression with larger effect sizes and higher significance were less likely to be due to hybridization artifact [11]. Thus, hybridization artifact will tend to inflate the number of weakly significant weakly differentially expressed genes. This tends to inflate Prob(*cis*-acting QTL) because this estimate is based on the complete distribution of p-values, including those which signal very slight differences in expression. An upward bias in

this quantity leads to an underestimate of the power to detect *cis*-acting QTL and, as described above, this shifts support toward the neutral model. Second, the role of hybridization artifact would be expected to increase as the locus-specific divergence increases, consistent with the neutral model of *cis*-acting QTL evolution. Thus, both because of its effect on the power estimate and its correlation with the locus-specific divergence, hybridization artifact would contribute support to the neutral model, but our analyses suggest that *cis*-acting QTL are not accumulating fast enough to be consistent with neutrality. Finally, we emphasize that our previous allele-specific expression quantitiative PCR experiments and comparative sequence analyses showing a strong signature of non-coding regulatory polymorphisms do not support a major role for hybridization artifact among genes with expression levels showing linkage to their own loci [3].

We next considered the possibility that major *trans*-acting QTL might bias our conclusions because of their widespread effects on many linked genes. Note that we attempt to correct for linked *trans*-acting QTL in our estimates of the power and false positive rate, but these calculations assume a uniform distribution of *trans*-acting regulatory loci which is not the case as can be seen from Figure 1. In order to explore this effect, we excluded chromosomes

II, III, V, VIII, XII, XIII, XIV, and XV which have major *trans*-acting QTL affecting a large number of genes throughout the genome and repeated the likelihood calculations. The estimated value of δ was 0.27 with the purifying selection model retaining statistical significance ($p = 2.0 \times 10^{-3}$), indicating that major *trans*-acting QTL are not significantly impacting the models.

If a large fraction of genes had expression levels too low to detect linkage to true underlying *cis*-acting QTL, this might lead to underestimation of the true underlying value of Prob(*cis*-acting QTL), causing us to over estimate the power to detect these QTL and hence to erroneously reject the neutral model. In order to test this possibility, we performed whole genome non-parametric linkage analysis on the expression levels of all 5067 genes using `R/qtl` and determined whether we were able to detect linkages elsewhere in the genome, even if we were unable to detect significant linkage to *cis*-acting QTL. We therefore sought to estimate π_0, the estimated proportion of null tests, for the most significant linkage in the entire genome for each gene expression trait. The multiple testing involved in obtaining a genome-wide maximum LOD scores means that twice the resulting non-parametric LOD score is no longer distributed as a χ^2_1 random variable. In order to obtain p-values associated with these genome-wide maximum LOD scores we

performed 10^5 whole genome scans on a permuted phenotype, collecting the genome-wide maximum LOD score from each permutation, and ranked each of the 5067 observed genome-wide maximum LOD scores in relationship to the 10^5 permuted data scores to obtain p-values. The estimated π_0 was approximately 0.0547 under this permutation based scheme for multiple testing correction. Note that inspection of Figure 1, in which 2368 out of 5067 genes show significant linkage, indicates that nearly all genes showing at least weak differential expression due to regulatory QTL is not unreasonable. Our ability to detect $1-\pi_0$ as large as 0.94 indicates that essentially all of the genes analyzed are expressed at levels sufficient to detect QTL somewhere in the yeast genome in a data set of the size of this one. Thus, the lower estimate of $1-\pi_0 \approx 0.49$ for linkage at the locus of the gene in question is due to the absence of *cis*-acting QTL at these loci rather than to low gene expression.

Finally, we considered the possibility that our purifying selection model might instead be capturing the fact that there are two classes of genes in the yeast genome with different numbers of regulatory sites. In this case, the probability that a gene shows *cis*-acting variation is

$$\text{Prob}(\textit{cis}\text{-acting QTL}) = 1 - (1-\delta)(1-e^{-\theta t n_1}) - \delta(1-e^{-\theta t n_2})$$

where n_1 is the average number of regulatory sites in genes of the first type

(which occur with frequency $1 - \delta$) and n_2 is the average number of regulatory sites in genes of the second type (which occur with frequency δ). Note that under the purifying selection model from the main text, δ fraction of genes have no mutable regulatory sites per gene ($n_2 = 0$) because purifying selection is assumed to have removed variation at regulatory sites in these genes. Under the model where n_2 is allowed to vary, the maximum likelihood estimates of δ, n_1, and n_2 were 0.24, 145, and 0.001. This model represents no significant improvement over the purifying selection model, and the parameter estimate of $n_2 = 0.001$ suggests that the data are best fit by a model in which δ fraction of genes have essentially have no mutable regulatory sites per gene. We believe that the most likely explanation for this small number of mutable regulatory sites is that purifying selection has eliminated regulatory polymorphism, but we cannot rigorously reject the possibility that such extreme variation in the number of regulatory sites per gene exists and that this accounts for the dampened rate of accumulation of *cis*-acting QTL.

Rare derived allele skew versus *cis*-regulatory effect size

In order to determine whether the signature of purifying select was stronger for genes with larger *cis*-acting fold changes in expression, we determined the magnitude of the allele frequency skew as a function of this quantity (Figure S2). The fold change in expression due to *cis*-regulatory polymorphism was estimated as $\frac{\max(x_{BY}, x_{RM})}{\min(x_{BY}, x_{RM})}$, where x_{BY} and x_{RM} were the mean expression level in segregants bearing the BY and RM alleles, respectively, at the marker closest to the gene in question. Sample sizes were 932, 908, 617, 367, and 228 genes with fold changes > 1, ≥ 1.05, ≥ 1.1, ≥ 1.15, and ≥ 1.2, respectively. The magnitude of the allele frequency skew became more variable at thresholds above 1.2, due to the small number of genes with fold changes in exceeding the threshold. Although the trend was not statistically significant (Fisher's exact $p = 0.24$ for promoter polymorphisms in genes with fold change ≥ 1.2 versus < 1.2), this analysis showed that the skew toward derived alleles increases for genes with larger *cis*-regulatory expression fold change. This suggests that regulatory alleles associated with larger expression changes tend to be more recent and presumably more efficiently

eliminated by natural selection.

Ancestral selection graph simulations

In order to interpret the skew in the allele frequency distribution of *cis*-acting regulatory polymorphisms, we performed simulations under the ancestral selection graph, an extension to the coalescent which incorporates natural selection [13, 14]. This allowed us to estimate what scaled selection coefficient would give rise to the observed skew toward rare derived alleles at regulatory sites relative to linked neutral sites. The demographic model is illustrated in the percolation diagram shown in Figure 5. At time $t = 37 \times N_e$ in the past, a common ancestral population representing both *S. cerevisiae* and *S. paradoxus* splits into two independently evolving populations. In the simulations, which are performed going backward in time, this corresponds to transferring all real and virtual ancestors in the *S. cerevisiae* and *S. paradoxus* populations into a common ancestral population. The value of t is derived from the maximum likelihood estimate (using `PAML` [15]) of the synonymous site divergence (approximately 0.38, thus $\frac{\theta}{2}(2t + 2) = 0.38$) between *S. cerevisiae* and *S. paradoxus*.

In addition to the selection model described in the main text, we varied whether transition mutations from the most strongly preferred type were associated with relatively smaller or larger changes in fitness. Figures S3 and S4 show results for $\sigma_T = 0$, $\sigma_G = 2N_es$, $\sigma_C = 2 \times 2N_es$, and $\sigma_A = 3 \times 2N_es$ and $\sigma_G = 0$, $\sigma_T = 2N_es$, $\sigma_C = 2 \times 2N_es$ and for $\sigma_A = 3 \times 2N_es$ with $N_es = 0.0, 0.2, 0.4, ...$, respectively. Under these models, the observed skew toward rare alleles is best fit when $2N_es = 1.2$ (95% CI 0.8-1.4) or 1.1 (95% CI 0.7-1.3), respectively. The relative rate of polymorphism between the sampled individuals representing BY and RM at the selected site compared to the neutral site was 73% or 72%, respectively. Note that while the best fitting value of N_es is somewhat less under these selection models than under the model considered in the main text, the estimated value of the key parameter of interest, ζ, the relative rate of polymorphism at the selected site, is very similar under all models.

We also considered models with fewer than four selective classes. Models in which $\sigma_T = \sigma_C = 0$ and $\sigma_G = \sigma_A = 2N_es$ showed no detectable skew toward rare alleles so we did not consider them further. Models in which $\sigma_T = \sigma_G = 0$ and $\sigma_C = \sigma_A = 2N_es$ yielded similar estimates of $2N_es$ (2.3) and ζ (0.71) as the four selective class models described above. Models in

which $\sigma_T = \sigma_C = \sigma_G = 0$ and $\sigma_A = 2N_e s$ showed the expected skew toward rare alleles but lead to increased rather than decreased levels of polymorphism between the two fitness classes for the range of selection coefficients which best fit the observed allele frequency skew. Given that we observe fewer rather than more *cis*-acting expression changes than expected under neutrality, we did not consider these models further.

References

[1] Brem RB, Yvert G, Clinton R, Kruglyak L (2002) Genetic dissection of transcriptional regulation in budding yeast. Science 296:752–755.

[2] Yvert G, Brem RB, Whittle J, Akey JM, Foss E, et al. (2003) *Trans*-acting regulatory variation in *Saccharomyces cerevisiae* and the role of transcription factors. Nature Genetics 35:57–64.

[3] Ronald J, Brem RB, Whittle J, Kruglyak L (2005) Local regulatory variation in *Saccharomyces cerevisiae*. PLoS Genetics 1:e25.

[4] R Development Core Team (2004) R: A language and environment for statistical computing. R Foundation for Statistical Computing, Vienna, Austria. URL `http://www.R-project.org`. ISBN 3-900051-07-0.

[5] Broman KW, Wu H, Sen S, Churchill GA (2003) `R/qtl`: QTL mapping in experimental crosses. Bioinformatics 19:889–890.

[6] Storey JD, Tibshirani R (2003) Statistical significance for genomewide studies. Proceedings of the National Academy of Sciences U S A 100:9440–9445.

[7] Brudno M, Do C, Cooper G, Kim MF, Davydov E, et al. (2003) `LAGAN` and `Multi-LAGAN`: efficient tools for large-scale multiple alignment of genomic DNA. Genome Research 13:721–731.

[8] Ruderfer DM, Pratt SC, Seidel HS, Kruglyak L (2006) Population genomic analysis of outcrossing and recombination in yeast. Nature Genetics 38:1077–1081.

[9] Hudson RR (2002) Generating samples under a Wright-Fisher neutral model of genetic variation. Bioinformatics 18:337–338.

[10] Rambaut A, Grassly NC (1997) `Seq-Gen`: An application for the Monte Carlo simulation of DNA sequence evolution along phylogenetic trees. Computer Applications in the Biosciences 13:235–238.

[11] Gilad Y, Rifkin SA, Bertone P, Gerstein M, White KP (2005) Multi-species microarrays reveal the effect of sequence divergence on gene expression profiles. Genome Research 15:674–680.

[12] Gilad Y, Oshlack A, Smyth GK, Speed TP, White KP (2006) Expression profiling in primates reveals a rapid evolution of human transcription factors. Nature 440:242–245.

[13] Neuhauser C, Krone SM (1997) The genealogy of samples in models with selection. Genetics 145:519–534.

[14] Krone SM, Neuhauser C (1997) Ancestral processes with selection. Theoretical Population Biology 51:210–237.

[15] Yang Z (1997) `PAML`: a program package for phylogenetic analysis by maximum likelihood. Computer Applications in Biosciences 13:555–556.
