# Supplementary figures and images for: The Evolution of Gene Expression QTL in Saccharomyces cerevisiae
Source: PLoS One. 2007 Aug 1;2(8):e678. doi: 10.1371/journal.pone.0000678 (PMC1925141; doi:10.1371/journal.pone.0000678)

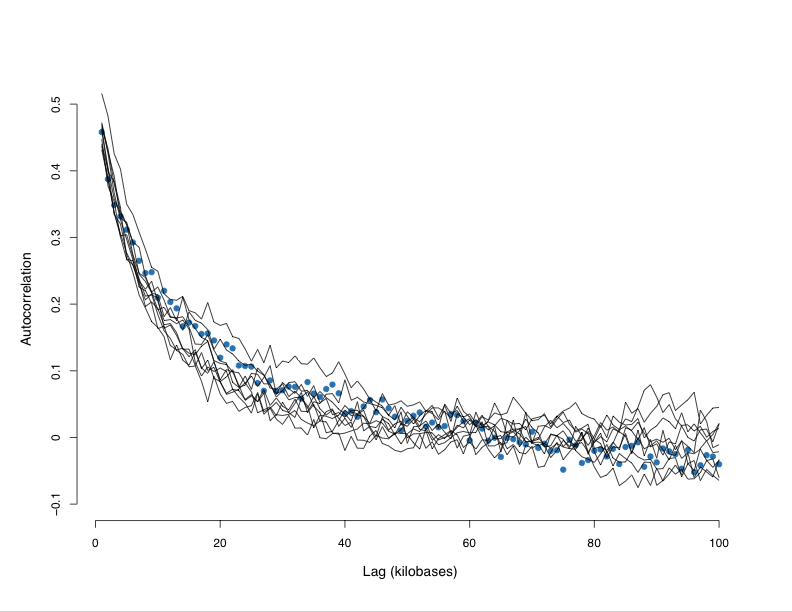

Supplement: Figure S1 — Observed synonymous site substitution rate autocorrelation function (blue points) and 10 realizations of the autocorrelation function from simulated yeast genomes (black lines). We imposed the same pattern of missing data on the simulated data as was present in the observed data (due to gaps, to low quality regions of the alignments, and to the absence of synonymous sites in intergenic regions and because of overlapping ORFs). In both the simulated and observed data the autocorrelation function was calculated for each chromosome and then averaged across the 16 chromosomes. (0.17 MB TIF) [file pone.0000678.s002.tif]

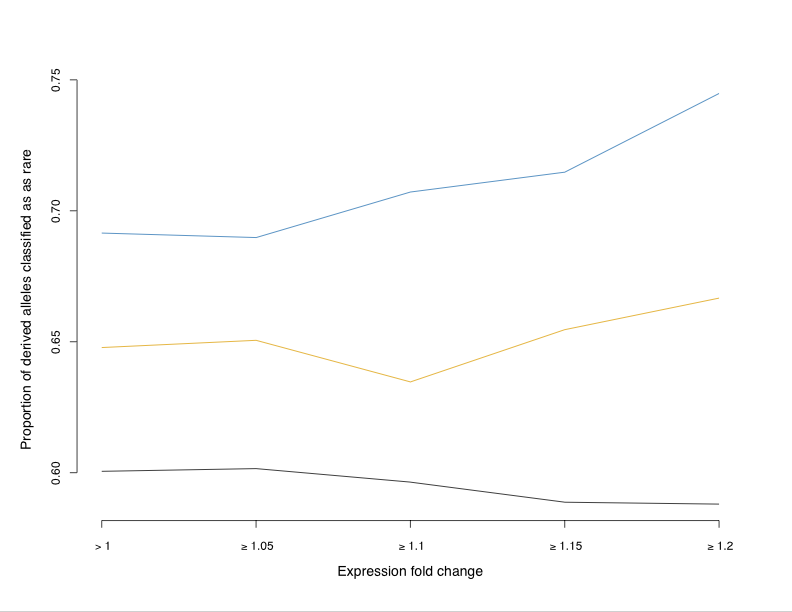

Supplement: Figure S2 — Magnitude of the skew toward derived alleles as a function of cis-regulatory effect size. The proportion of derived alleles classified as rare are shown for synonymous polymorphisms (black), promoter polymorphisms (blue), and 3' UTR polymorphisms (orange) in genes with cis-regulatory variation. (0.11 MB TIF) [file pone.0000678.s003.tif]

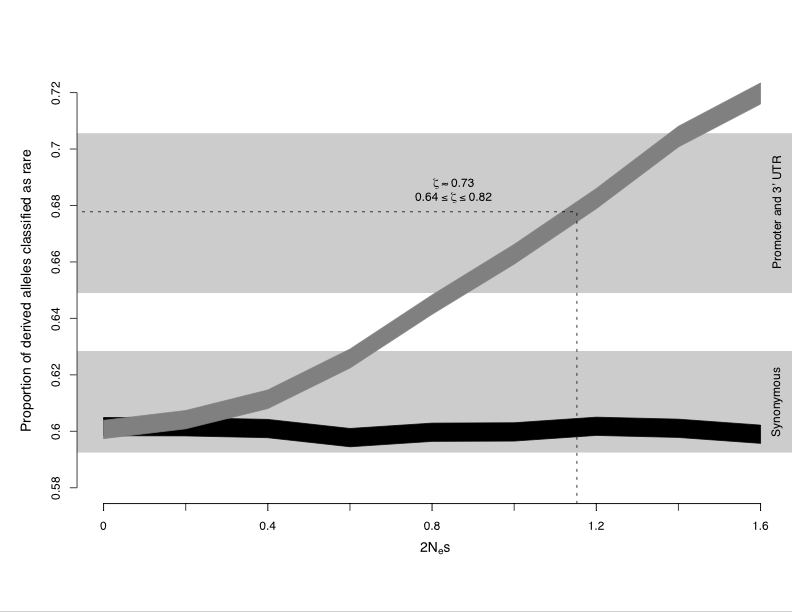

Supplement: Figure S3 — Strength of purifying selection against cis-acting regulatory changes with ΣT = 0, ΣG = 2Nes, ΣC = 2×2Nes, and ΣA = 3×2Nes. The relative rate of substitution at the selected site relative to the linked neutral site, denoted by ζ, is indicated along with the 95% CI. (0.85 MB TIF) [file pone.0000678.s004.tif]

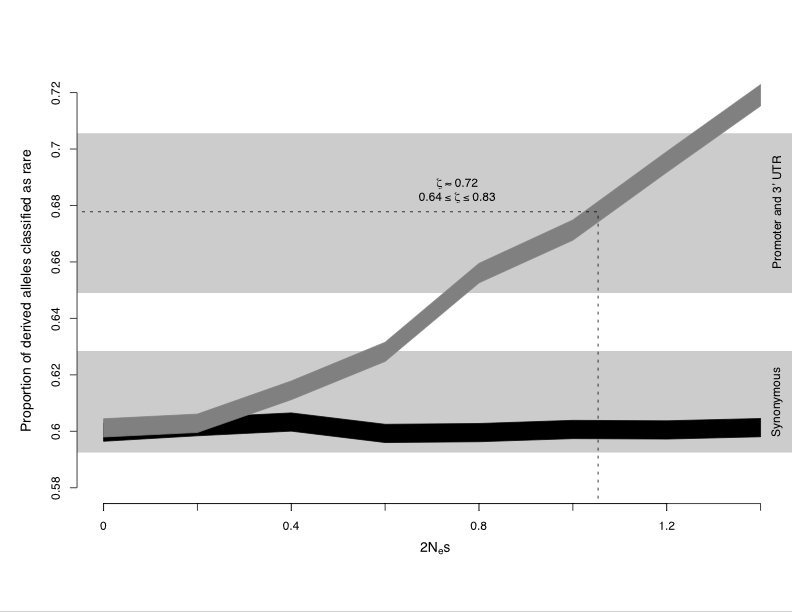

Supplement: Figure S4 — Strength of purifying selection against cis-acting regulatory changes with ΣG = 0, ΣT = 2Nes, ΣC = 2×2Nes, and ΣA = 3×2Nes. The relative rate of substitution at the selected site relative to the linked neutral site, denoted by ζ, is indicated along with the 95% CI. (0.84 MB TIF) [file pone.0000678.s005.tif]
